# Identification of genetic alterations associated with primary resistance to EGFR-TKIs in advanced non-small-cell lung cancer patients with EGFR sensitive mutations

**DOI:** 10.1186/s40880-019-0354-z

**Published:** 2019-03-02

**Authors:** Fang Wang, Xia-Yao Diao, Xiao Zhang, Qiong Shao, Yan-Fen Feng, Xin An, Hai-Yun Wang

**Affiliations:** 1 State Key Laboratory of Oncology in South China, Collaborative Innovation Center for Cancer Medicine, Sun Yat-sen University Cancer Center, Guangzhou, 510060 Guangdong P.R. China; 2 Department of Molecular Diagnostics, Sun Yat-sen University Cancer Center, No. 651 Dongfeng Road East, Guangzhou, 510060 Guangdong P.R. China; 3 Department of Urology, Sun Yat-sen Memorial Hospital, Guangzhou, 510120 Guangdong P.R. China; 4 Department of Pathology, Sun Yat-sen University Cancer Center, Guangzhou, 510060 Guangdong P.R. China; 5 Department of Medical Oncology, Sun Yat-sen University Cancer Center, Guangzhou, 510060 Guangdong P.R. China

**Keywords:** Epidermal growth factor receptor, Tyrosine kinase inhibitors, Resistance, Non-small-cell lung cancer

## Abstract

**Background:**

Identification of activated epidermal growth factor receptor (*EGFR*) mutations and application of EGFR-tyrosine kinase inhibitors (EGFR-TKIs) have greatly changed the therapeutic strategies of non-small-cell lung cancer (NSCLC). However, the long-term efficacy of EGFR-TKI therapy is limited due to the development of drug resistance. The aim of this study was to investigate the correlation between the aberrant alterations of 8 driver genes and the primary resistance to EGFR-TKIs in advanced NSCLC patients with activated EGFR mutations.

**Methods:**

We retrospectively reviewed the clinical data from 416 patients with stage III/IV or recurrent NSCLC who received an initial EGFR-TKI treatment, from April 2004 and March 2011, at the Sun Yat-sen University Cancer Center. Several genetic alterations associated with the efficacy of EGFR-TKIs, including the alterations in *BIM*, *ALK*, *KRAS*, *PIK3CA*, *PTEN*, *MET*, *IGF1R*, and *ROS1*, were detected by the routine clinical technologies. The progression-free survival (PFS) and overall survival (OS) were compared between different groups using Kaplan–Meier survival analysis with the log-rank test. A Cox regression model was used to estimate multivariable-adjusted hazard ratios (HRs) and their 95% confidence intervals (95% CIs) associated with the PFS and OS.

**Results:**

Among the investigated patients, 169 NSCLC patients harbored EGFR-sensitive mutations. EGFR-mutant patients having PTEN deletion had a shorter PFS and OS than those with intact PTEN (*P* = 0.003 for PFS, and *P* = 0.034 for OS). In the combined molecular analysis of EGFR signaling pathway and resistance genes, we found that EGFR-mutant patients coexisted with aberrant alterations in EGFR signaling pathway and those having resistant genes had a statistically poorer PFS than those without such alterations (*P* < 0.001). A Cox proportional regression model determined that PTEN deletion (HR = 4.29,95% CI = 1.72–10.70) and low PTEN expression (HR = 1.96, 95% CI = 1.22–3.13), MET FISH + (HR = 2.83,95% CI = 1.37–5.86) were independent predictors for PFS in patients with EGFR-TKI treatment after adjustment for multiple factor.

**Conclusions:**

We determined that the coexistence of genetic alterations in cancer genes may explain primary resistance to EGFR-TKIs.

**Electronic supplementary material:**

The online version of this article (10.1186/s40880-019-0354-z) contains supplementary material, which is available to authorized users.

## Introduction

Several large scale phase III clinical trials have confirmed that activating epidermal growth factor receptor (*EGFR*) mutations (mutations in exons 18-21) in the treatment of patients with advanced non-small-cell lung cancer (NSCLC) increases the tumor sensitivity towards EGFR-tyrosine kinase inhibitors (EGFR-TKIs) such as gefitinib, afatinib and erlotinib [1–5]. However, drug resistance, comprised of primary and acquired resistance, greatly limits the long-term efficacy of EGFR-TKI therapies.

The mechanisms of acquired resistance to EGFR-TKIs mainly include the acquisition of the *EGFR* T790M mutation [6] and the amplification of the *MET* proto-oncogene, receptor tyrosine kinase (*MET*) [7]. However, 20%–30% of patients develop primary resistance to EGFR-TKIs despite having tumors that harbor active *EGFR* mutations [1, 8, 9]. Several studies have reported the mechanisms of primary resistance to EGFR-TKIs, including the acquisition of Kirsten rate sarcoma viral oncogene homolog (*KRAS*) mutations and phosphatase and tensin homolog (*PTEN*) loss [10, 11]. Additionally, a previous study reported that 27.3% (6/22 cases) of NSCLC patients exhibited a decreased expression of the kappa-light-chain-enhancer of activated B cell (*IκB*), 9.1% (2/22 cases) had increased expression of insulin-like growth factor 1 receptor (*IGF1R*), 18.2% (4/22 cases) had Bcl-2 like protein 11 (*BIM*) polymorphism, and 22.7% (5/22 cases) had AXL expression [12]. However, none of the above molecular alterations could explain the primary resistance to EGFR-TKIs in the majority of cases, and most of these mechanisms have not yet been clinically validated. Additionally, the incidence and clinicopathological characteristics of major primary resistance mutations in terms of EGFR-TKIs response and outcome in NSCLCs are well established.

Herein, we used clinical routine technologies to determine the alteration status of 8 major driver genes, including the anaplastic lymphoma kinase (*ALK*), *KRAS*, phosphatidylinositol-4,5-bisphosphate 3-kinase catalytic subunit alpha (*PIK3CA*), *BIM*, *PTEN*, *MET*, *IGF1R* and ROS proto-oncogene 1 (*ROS1*) in advanced NSCLC patients. In addition, the effect of each gene and/or their combined alterations on EGFR-TKIs clinical responses and survival were investigated in EGFR-mutant NSCLC patients receiving EGFR-TKIs treatment.

## Materials and methods

### Patient selection

A total of 416 patients hospitalized at the Sun Yat-sen University Cancer Center (Guangzhou, China) with histologically confirmed stage IIIb, stage IV, or recurrent NSCLC receiving gefitinib or erlotinib treatment from April 2004 to March 2011 were investigated. The characteristics of each patient was documented by a retrospective chart review, which included age at diagnosis, gender, smoking status, clinical stage, dates of diagnosis and death, disease recurrence, Eastern Cooperative Oncology Group (ECOG) performance status at the start of treatment with EGFR-TKI, the number of previous chemotherapy regimens received, the EGFR-TKI administered (gefitinib or erlotinib), and subsequent treatment after tumor progression. Patients who have never smoked a cigarette or who have smoked fewer than 100 cigarettes in their entire lifetime were defined as non-smokers. Clinical stage was calculated based on the 2009 revised international staging system for lung cancer by the Union for International Cancer Control (UICC) [13]. The inclusion criteria were as follows: (1) sufficient tumor tissue obtained by surgical resection, biopsy or puncture from primary or metastatic tumors at the time of initial diagnosis; (2) the presence of at least one measurable lesion according to the Response Evaluation Criteria In Solid Tumors (RECIST version 1.0) [14]; and (3) complete follow-up information (at least one evaluation before disease progression, more than 3 months after follow-up, or upon death). Patients were excluded if they had brain metastases or other primary cancers that were diagnosed either before or after the diagnosis of NSCLC. The study was approved by the Research Ethics Committee (No. B2018-157-01) of the Sun Yat-sen University Cancer Center.

### DNA extraction and mutation analysis

NSCLC tissues with histological control for the presence of tumor cells (> 70%) were obtained at surgery by trimming the normal and necrotic tissues. DNA from formalin-fixed, paraffin-embedded tissues was isolated using the QIAamp DNA FFPE Tissue kit (QIAGEN, Hilden, Germany)### according to the manufacturer’s instruction. Twenty-two mutations of *EGFR* in exons 18-21 were screened by using the Surplex^®^ EGFR Mutation Kit (Surexam Bio-Tech, Guangzhou, China) as previously described [15]. *KRAS* and *PIK3CA* mutation status were detected by using the OncoCarta Panel (version 1.0; Sequenom Inc., San Diego, CA, USA). Mutation data were analyzed using the MassARRAY Typer software (version 4.0; Sequenom Inc.), and a cutoff mutation frequency of 1% was applied. *BIM* deletion was detected by quantitative real-time PCR using selected primers and minor groove binder (MGB) probes. The PCR assay was performed using the ABI 7500 (Applied Biosystems, Foster City, CA, USA). Genomic DNA (20 ng) was amplified in a 25 μL reaction. Each sample was assayed in triplicate with positive and negative controls. The primers and probes used for this step are as follows: the forward primer: 5′-GCTCTGTCTTCATAGGCTTCAG-3′ for both wild-type and mutant *BIM*; the reverse primer: 5′-GAGGTTCTTTCCAATTCTATAACAT-3′ and the probe: 5′-FAM-AAAGGCCTGCCTGATTTACCTC-BHQ1-3′ for wild-type *BIM*; and the reverse primer: 5′-TGTTGGTGGGAATGTAAAATG-3′ and the probe: 5′-HEX-CCTCTATGGAGAACAGTGATTTACCTC-BHQ1-3′ for mutant *BIM*.

### Copy number analysis using fluorescence in situ hybridization (FISH)

The gene copy numbers of *EGFR*, *PTEN*, *MET* and *IGF1R* in NSCLC cells were determined by FISH using the formalin-fixed, paraffin embedded tissues of lung resected specimens. Multi-color FISH assays were performed as described previously [18], using an EGFR/chromosome 7 centromere probe (Vysis, Abbott Laboratories, IL, USA), a PTEN/chromosome 10 centromere probe (Kreatech Diagnostics, Amsterdam, Netherlands), a MET/chromosome 7 centromere probe (Kreatech Diagnostics), and an IGF1R/chromosome 15 centromere probe (Kreatech Diagnostics). Analysis was performed using the Olympus BX61 microscope (Olympus, Tokyo, Japan). For documentation, images were captured using a charge-coupled device camera (Olympus, Tokyo, Japan) and merged using the BioView Automated Imaging Analysis System (BioView Ltd, Israel). The scoring was carried out in 50 non-overlapping tumor cell nuclei per patient from four representative tumor areas. Based on the University of Colorado Cancer Center (UCCC) criteria [15], the gene copy numbers for *EGFR* and *IGF1R* were classified as FISH (+) if they displayed gene amplification or high polysomy and FISH (−) if they displayed normal disomy, trisomy or low polysomy. Alternatively, *MET* gene status was determined using the Cappuzzo scoring system as previously described [16]. FISH (+) *MET* encompassed MET/CEP7 ratio ≥ 2 or MET signals ≥ 5 per cell otherwise, it was considered as MET FISH (−). The FISH analysis and scoring of *PTEN* were performed as previously described [17]. The gene copy numbers for *PTEN* were classified as FISH (+) if they displayed gene hemizygous/homozygous deletion or whole chromosome 10 deletion and FISH (−) if they displayed normal disomy or polysomy.

### Immunohistochemistry (IHC)

IHC evaluations on 4-μM tissue sections were performed on a BenchMark XT automated slide processing system (Ventana, Tucson, AZ, USA) with the Ventana SP44 anti-MET antibody and Ventana G11 anti-IGF1R antibody (Ventana Medical Systems, Tucson, AZ, USA) according to the manufacturer’s protocol, and the mouse monoclonal anti-PTEN (clone 6H2.1; Dako, Glostrup, Denmark) according to the protocol published previously [18]. Histoscore (H-score) was calculated by a semi-quantitative assessment of both the staining intensity (graded as: 0, no stain; 1, weak; 2, moderate; or 3, strong using adjacent normal tissue as the median) and the percentage of positive cells (0–100%) according to the criteria of the hybrid scoring system [19]. High MET and IGF1R expression were defined as having an H-score > 100 (H-score [+]). Moreover, additional evaluation of MET expression was also performed according to the OAM4558g MetMAb phase II trial which defined the scoring criteria for MAb [+], defined as having ≥ 50% of tumor cells with moderate or strong membranous staining [20]. Low PTEN expression was defined as having ≥ 10% of tumor cells with weak or without cytoplasmic staining. Adjacent normal epithelium within the tissue section was used as a positive control [17]. Histopathology analysis was performed by two pathologists who were blinded of the patients’ clinical characteristics and molecular variables.

### Gene rearrangement analysis using FISH

FISH was performed on unstained 4-µm formalin-fixed and paraffin embedded NSCLC tissue sections with the use of an ALK or ROS1 break-apart rearrangement probe set (Vysis) according to a previously published protocol [21].

Signal analysis was performed using the Olympus BX61 microscope (Olympus, Japan) equipped with a triple-pass filter (DAPI/Green/Orange, Vysis), a charge-coupled device camera (Olympus, Japan) and merged using the BioView Automated Imaging Analysis System (BioView Ltd, Israel) The analysis was carried out in 50 non-overlapping tumor cell nuclei per patient. According to the manufacturer’s instruction, tumor tissues were considered as ALK-FISH (+) or ROS1-FISH (+) (ALK-rearranged or ROS1-rearranged) if ≥ 15% of the tumor cells showed split signals. Otherwise, the samples were considered as ALK-FISH (−) or ROS1-FISH (−). FISH analysis was performed by two pathologists who were blinded of the patients’ clinical characteristics and molecular variables.

### Follow-up

Gefitinib or erlotinib was orally administered at a daily dose of 250 mg or 150 mg, respectively until disease progression, intolerable toxicity, or patient refusal. Clinical response was assessed every 3–10 weeks by radiologic examination (computed tomography or magnetic resonance imaging). Brain magnetic resonance imaging or radionuclide bone scan were added when brain or bone metastasis was suspected. The therapeutic effectiveness was evaluated based on the RECIST version 1.0 and the therapeutic response was categorized into these following categories: progressive disease (PD), stable disease (SD), partial response (PR), and complete response (CR). Patients exhibiting CR and PR were considered objective response to EGFR-TKIs, while patients with CR, PR, and SD were regarded as disease-control patients.

Follow-up information was obtained from the in- or out-patient medical records and telephone interviews every 3 months after EGFR-TKIs treatment until March 20, 2014, radiographically identified tumor progression or death. Progression-free survival (PFS) was calculated from the time of the first EGFR-TKI treatment to the time of disease progression according to the RECIST version 1.0, or unacceptable toxic effects. Overall survival (OS) was calculated from the time of first EGFR-TKI treatment to the time of the patient’s death from any cause or last contact.

### Statistical analysis

Categorical variables were compared between the EGFR mutant group and wild-type group using the Chi squared (χ^2^) test and Fisher’s exact test. Objective response rate (ORR) and disease control rate (DCR) were evaluated using the χ^2^ test. PFS and OS were compared between different groups using the Kaplan–Meier survival analysis with the log-rank test. Cox proportional hazards regression analyses were used to evaluate the independent predictive factors of each biological and clinical feature associated with survival. All statistical analyses were performed with the SPSS software version 19.0 for Windows (IBM, Armonk, NY, USA). A two-sided *P* value less than 0.05 was considered significant.

## Results

### Patient characteristics

A total of 416 NSCLC patients treated with EGFR-TKIs between April 2004 and March 2011 were retrospectively investigated. Patient characteristics are summarized in Table 1. In the entire cohort, 336 (80.8%) patients were histologically diagnosed as lung adenocarcinoma (ADC), 269 (64.7%) were non-smokers, 169 (40.6%) harbored EGFR mutations, and 167 (40.1%) were EGFR-FISH (+). The most frequent mutations of EGFR were exon 19 deletions (89 cases, 52.7%) and L858R point mutation (73 cases, 43.2%). Among all the patients enrolled, 93 (22.4%) achieved an objective response, 158 (38.0%) had SD, and 161 (38.7%) had PD. Patients with EGFR mutations and EGFR-FISH (+) had a significant response to EGFR-TKIs compared with those harboring wild-type EGFR and EGFR-FISH (−) (Table 1; *P* < 0.001).

**Table 1:** Clinical characteristics of 416 NSCLC patients treated with EGFR-TKIs

Variables	Patients		EGFR mutation			EGFR copy number		
	n = 416		Mutant	Wild		FISH (+)	FISH (−)	
	No.	%	n = 169	n = 247	*P* value	n = 167	n = 249	*P* value
Age groups								
≤ 60	264	63.5	108 (63.9)	156 (63.2)	0.918	108 (64.7)	156 (62.7)	0.680
> 60	152	36.5	61 (36.1)	91 (36.8)		59 (35.3)	93 (37.3)	
Gender								
Male	253	60.8	86 (50.9)	167 (67.6)	< 0.001	92 (55.1)	161 (64.7)	0.052
Female	163	39.2	83 (49.1)	80 (32.4)		75 (44.9)	88 (35.3)	
Smoking status								
Never-smoker	269	64.7	129 (76.3)	140 (56.7)	< 0.001	116 (69.5)	155 (62.2)	0.142
Smoker	147	35.3	40 (23.7)	107 (43.3)		51 (30.5)	94 (37.8)	
Histology								
ADC	336	80.8	154 (91.1)	182 (73.7)	< 0.001	138 (82.6)	198 (79.5)	0.449
Non-ADC	80	19.2	15 (8.9)	65 (26.3)		29 (17.4)	51 (20.5)	
Differentiation								
High to medium	185	44.5	92 (54.4)	93 (37.7)	< 0.001	85 (50.9)	100 (40.2)	0.035
Low	231	55.5	77 (45.6)	154 (62.3)		82 (49.1)	149 (59.8)	
Disease stage								
Recurrent	128	30.8	55 (32.5)	73 (29.6)	0.127	51 (30.5)	77 (30.9)	0.926
IIIb	73	17.5	22 (13.0)	51 (20.6)		28 (16.8)	45 (18.1)	
IV	215	51.7	92 (54.4)	123 (49.8)		88 (52.7)	127 (51.0)	
Clinical response								
CR + PR	93	22.4	68 (40.2)	25 (10.1)	< 0.001	53 (31.7)	40 (16.1)	< 0.001
SD	158	38.0	83 (49.1)	75 (30.4)		79 (47.3)	79 (31.7)	
PD	161	38.7	14 (8.3)	147 (59.5)		32 (19.2)	129 (51.8)	
Unknown	4	1.0	4 (2.4)	0 (0.0)		3 (1.8)	1 (0.4)	
Line of EGFR-TKIs								
First	104	25.0	62 (36.7)	42 (17.0)	< 0.001	48 (28.7)	56 (22.5)	< 0.001
Second	217	52.2	107 (63.3)	110 (44.5)		97 (58.1)	120 (48.2)	
Higher	95	22.8	0 (0.0)	95 (38.5)		22 (13.2)	73 (29.3)	

Non-adenocarcinoma included squamous-cell carcinoma (n = 58), adenosquamous cell carcinoma (n = 20), and other histology (n = 2)

*ADC* adenocarcinoma, *FISH* fluorescent in situ hybridization, *CR* complete remission, *PR* partial remission, *SD* stable disease, *PD* progressive disease, *TKI*, tyrosine kinase inhibitor

We performed the MassARRAY, quantitative real-time PCR, FISH and IHC analyses to evaluate the status of KRAS, PIK3CA, ALK, BIM, PTEN, MET, IGF1R and ROS1 gene in the whole cohort. All patients enrolled were ROS1-FISH (−). Representative images of IHC staining and FISH analysis for ALK, PTEN, MET and IGFR1 in tumor tissues are shown in Additional file 1: Figure S1. The association between the clinicopathological features and the genetic alterations of these genes are summarized in Additional file 2: Table S1. The survival analysis of each gene alteration is presented in Additional file 3: Figure S2 and Additional file 4: Figure S3. The last follow-up date was March 20, 2014, and the median follow-up time for this study was 19.5 months (range, 2.9-80.1 months). At the time of the last follow-up date, 19 (4.6%) patients were still being treated with EGFR-TKIs, and 249 (59.9%) had succumbed to their disease.

### Univariate and multivariate analysis

The univariate and multivariate associations of the clinicopathological parameters and the alterations of each gene with PFS and OS in the 416 NSCLC patients are shown in Table 2. After multivariate adjustment, the female patients (HR = 0.56, 95% CI = 0.42-0.47), EGFR mutation (HR = 0.54, 95% CI = 0.42–0.70), FISH + (HR = 0.67, 95% CI = 0.54–0.84), and ECOG PS 0/1 (HR = 0.71, 95% = 0.52–0.95) were confirmed to be statistically significant as favorable factors for longer PFS in NSCLC patients treated with EGFR-TKIs, whereas MET copy number gain (HR = 1.51, 95% CI = 1.07–2.13) was statistically significant predictors of shorter PFS (Table 2).

**Table 2:** Univariate and multivariate associations of EGFR mutation and FISH status, clinicopathological characteristics, and status of other seven genes with PFS and OS in the total of 416 NSCLC patients

Variable	PFS						OS					
	Univariate			Multivariate			Univariate			Multivariate		
	HR	95% CI	*P*	HR	95% CI	*P*	HR	95% CI	*P*	HR	95% CI	*P*
Age												
≤ 60	1						1					
> 60	1.01	0.82–1.25	0.913				1.21	0.94–1.57	0.145			
Gender												
Male	1			1			1			1		
Female	0.59	0.48–0.73	< 0.001	0.56	0.42–0.74	< 0.001	0.59	0.45–0.76	< 0.001	0.65	0.48–0.94	0.006
Smoking status												
Smoking	1			1			1			1		
No smoking	0.60	0.48–0.74	< 0.001	0.64	0.50–0.81	< 0.001	0.62	0.48–0.80	< 0.001	0.79	0.58–1.06	0.113
Histology												
Non-ADC	1			1			1					
ADC	0.67	0.52–0.86	0.002	0.84	0.64–1.10	0.203	0.78	0.58–1.07	0.122			
Differentiation												
High to medium	1			1			1					
Low	1.36	1.11–1.68	0.004	0.89	0.72–1.18	0.299	1.12	0.87–1.44	0.363			
Stage												
Recurrent	1						1					
IIIB and IV	0.88	0.70–1.10	0.254				0.83	0.63–1.09	0.183			
Line of TKI therapy												
2, 3	1			1			1			1		
1	0.68	0.53–0.87	0.002	0.75	0.58–0.97	0.027	0.67	0.50–0.90	0.009	0.78	0.57–1.05	0.103
ECOG PS												
2	1			1			1			1		
0, 1	0.71	0.52–0.95	0.023	0.73	0.54–0.99	0.043	1.71	1.17–2.48	0.004	0.62	0.43–0.90	0.011
EGFR mutation												
Mt	1			1			1			1		
Wt	0.38	0.31–0.47	< 0.001	0.54	0.42–0.70	< 0.001	0.50	0.38–0.65	< 0.001	0.63	0.47–0.85	0.002
EGFR copy number												
FISH−	1			1			1			1		
FISH+	0.54	0.43–0.67	< 0.001	0.67	0.54–0.84	0.001	0.60	0.46–0.78	< 0.001	0.77	0.58–1.02	0.068
ALK												
Wt	1			1			1			1		
Apart	2.06	1.41–3.02	< 0.001	1.41	0.93–2.16	0.105	1.59	1.00–2.51	0.047	1.34	0.82–2.17	0.240
BIM												
Wt	1						1					
Deletion	0.89	0.58–1.36	0.596				1.05	0.64–1.71	0.856			
KRAS mutation												
Wt	1			1			1					
Mt	1.99	1.33–2.99	0.001	1.21	0.79–1.84	0.386	1.55	0.97–2.47	0.070			
PIK3CA mutation												
wt	1						1					
mt	1.18	0.61–2.29	0.619				1.22	0.60–2.47	0.583			
PTEN copy number												
Intact	1						1					
Deletion	1.54	0.99–2.39	0.058				1.06	0.64–1.77	0.811			
PTEN expression												
Normal	1			1			1					
Low expression	1.37	1.08–1.74	0.011	1.10	0.86–1.42	0.439	1.12	0.84–1.50	0.448			
MET copy number												
FISH−	1			1			1			1		
FISH+	1.87	1.35–2.60	< 0.001	1.51	1.07–2.13	0.019	1.64	1.14–2.37	0.008	1.47	1.01–2.13	0.045
MET expression												
MAb−	1						1					
MAb+	1.01	0.79–1.28	0.955				1.18	0.87–1.59	0.283			
MET expression												
H-score−	1						1					
H-score+	1.14	0.93–1.40	0.217				0.97	0.75–1.25	0.805			
IGF1R copy number												
FISH−	1			1			1					
FISH+	1.40	1.05–1.87	0.019	1.07	0.78–1.46	0.688	1.10	0.77–1.57	0.619			
IGF1R expression												
IHC−	1						1					
IHC+	1.26	0.99–1.61	0.062				1.05	0.78–1.40	0.770			

*ADC* adenocarcinoma, *ECOG* Eastern cooperative oncology group, *TKI* tyrosine kinase inhibitors, *FISH* fluorescent in situ hybridization, *HR* hazard ratio, *CI* confidence interval, *PFS* progression-free survival, *OS* overall survival

### Combined analysis of molecular driver genes and survival analysis

**Fig. 1:**
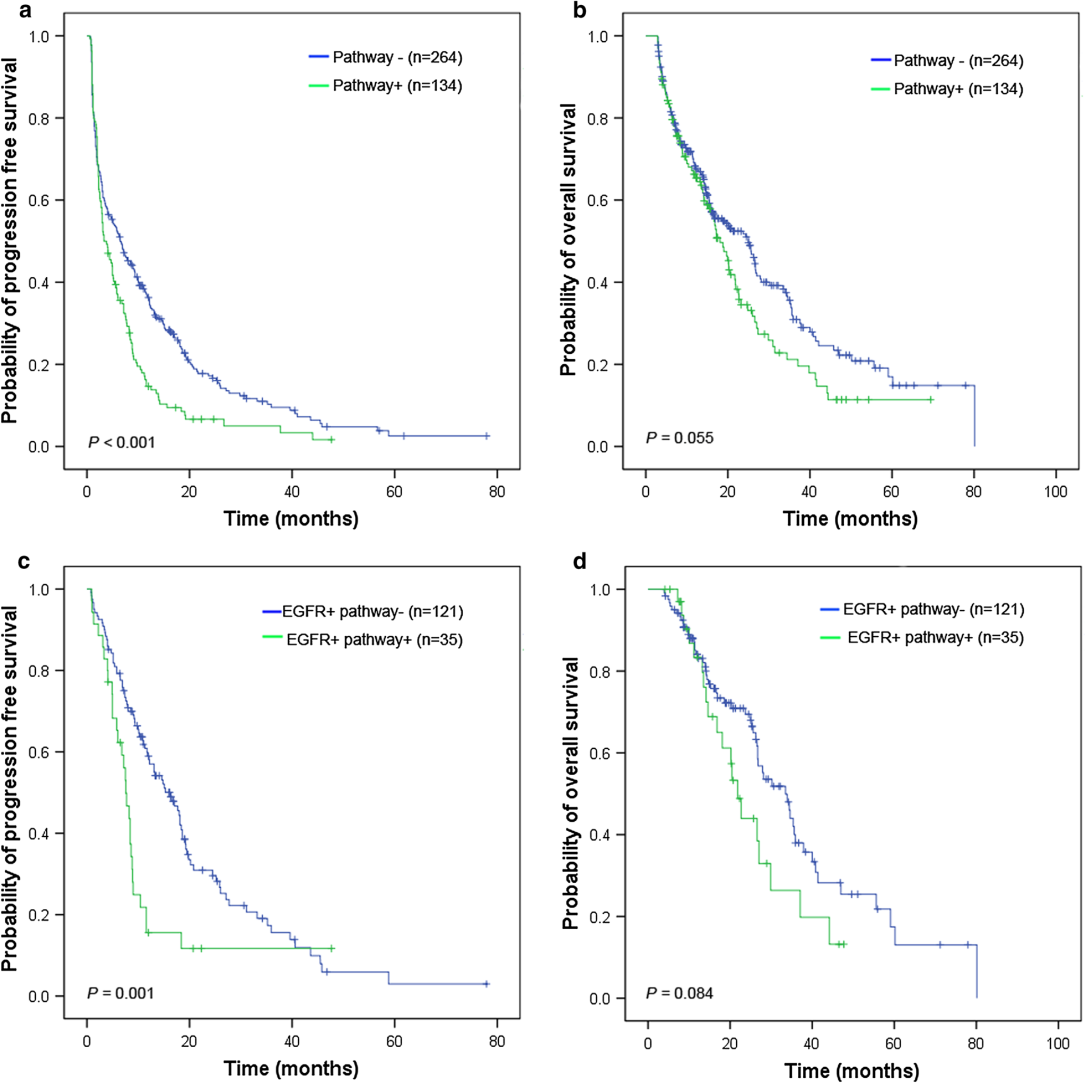
Kaplan-Meier curves of the progression-free survival (PFS) and overall survival (OS) for NSCLC patients with EGFR signaling pathway alterations. PFS (**a**) and OS (**b**) were analyzed in the 416 NSCLC patients, according to the EGFR signaling pathway alterations. In the 156 NSCLC patients with EGFR mutation, PFS (**c**) and OS (**d**) were analyzed according to the EGFR signaling pathway alterations. The survival rates were compared using the log-rank test. *NSCLC* non-small cell lung cancer, *PFS* progression-free survival, *OS* overall survival

To clarify the understanding of the effect of aberrant alterations in multiple genes on PFS and OS, we categorized the selected genes into two subgroups according to biological knowledge regarding the EGFR signaling pathway and the resistance genes (RG). RAS-RAF-MARK and PI3K/AKT/mTOR are two important pathways in EGFR signaling regulation, and we considered any changes in KRAS, PIK3CA, and PTEN as alterations in the EGFR signaling pathway; which included mutations, gain/loss in copy number, and demonstration of high/low expression [22]. EGFR signaling pathway alterations were observed in 35 (20.7%) and 99 (40.1%) NSCLC patients based on their EGFR status (mutation and wild-type, respectively). The Kaplan–Meier analysis showed that patients with aberrant alterations in the EGFR signaling pathway had inferior PFS compared to those without EGFR signaling pathway changes (3.3 months vs. 6.5 months, HR = 1.50, 95% CI = 1.20–1.97, Fig. 1a). Subsequently, a survival analysis demonstrated that mutant-EGFR NSCLC patients harboring EGFR signaling pathway alterations had poorer PFS than those without EGFR signaling pathway alterations (7.6 months vs. 16.3 months, HR = 2.07, 95% CI = 1.35–3.18, Fig. 1c), but not for OS analysis (21.8 months vs. 33.5 months, HR = 1.57,95% CI = 0.94–2.63, Fig. 1d).

**Fig. 2:**
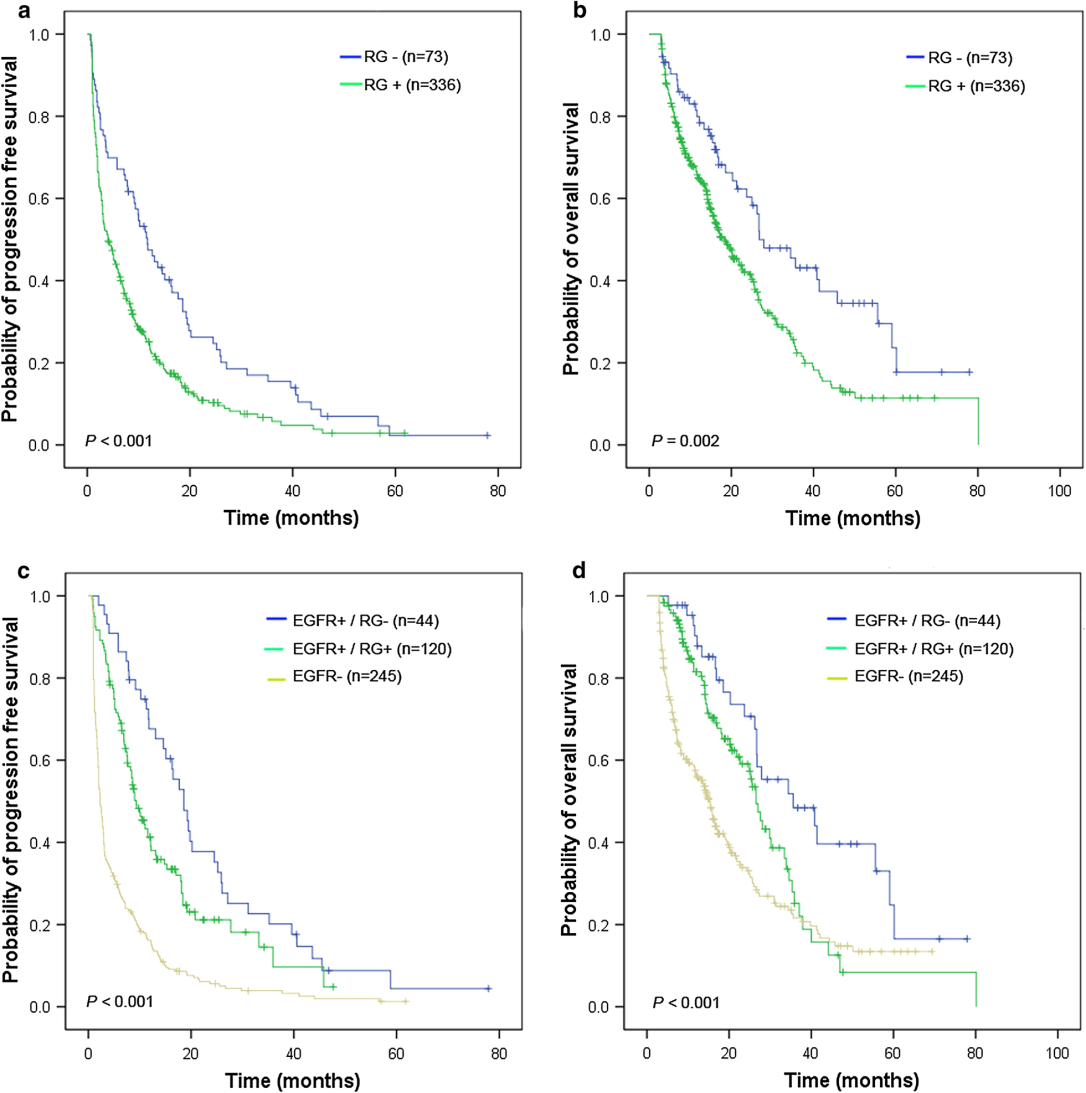
Kaplan-Meier curves of the progression-free survival (PFS) and overall survival (OS) for NSCLC patients with RG alterations. PFS (**a**) and OS (**b**) were analyzed in the 416 NSCLC patients, according to the RG alterations. In the subgroups of NSCLC patients that were EGFR+/RG−, EGFR+/RG+, EGFR−, PFS (**c**) and OS (**d**) were analyzed. The survival rates were compared using the log-rank test. *NSCLC* non-small cell lung cancer, *PFS* progression-free survival, *OS* overall survival, *RG* resistance gene

Seven driver genes, ALK, KRAS, BIM, PIK3CA, MET, IGF1R, and PTEN, with any changes involving mutation, copy number and expression have already been identified as the aberrant alterations in resistance genes (RG) in previous studies [12, 23–25]. Of the 416 NSCLC patients, 336 patients (80.7%) harbored at least one RG alteration. Overall, the obtained ORR and DCR were lower in the subgroups with RG alterations than in those without the RG alterations (18.3% vs. 41.7%, *P* < 0.001 and 58.0% vs. 73.6%, *P* = 0.016, respectively). In addition, the ORR was higher in the EGFR(+)/RG(−) subgroup than in those with EGFR(+)/RG(+) and EGFR(−) (60.5% vs. 34.2% vs. 10.2%, *P* < 0.05). NSCLC patients with RG alterations had shorter PFS and OS than those without RG alterations (*P* = 0.001, Fig. 2a and *P* = 0.002, Fig. 2b). Among the subgroups with EGFR(+)/RG(−), EGFR(+)/RG(+), and EGFR(−), there were statistically significant differences in the observed PFS and OS (18.6 months, 9.3 months and 2.3 months for PFS, respectively; and 35.6 months, 26.6 months and 15.4 months for OS, respectively; Fig. 2c, d, *P* < 0.005). It was further demonstrated that RG with/without aberrant alterations had no effect on PFS and OS in EGFR-wild-type NSCLC patients (data not shown). However, when considering EGFR FISH(+) as a favorable factor, EGFR FISH(+) NSCLC patients integrated with EGFR wild/RG(+) had longer PFS than those with EGFR FISH(−) (3.3 months vs. 2.0 months, HR = 0.60, 95% CI = 0.43–0.82), and this improved trend continued to OS (16.6 months vs. 13.9 months, HR = 0.64, 95% CI = 0.43–0.95).

### EGFR mutation patients had poorer survival when co-mutated with PTEN/MET aberrant changes

**Fig. 3:**
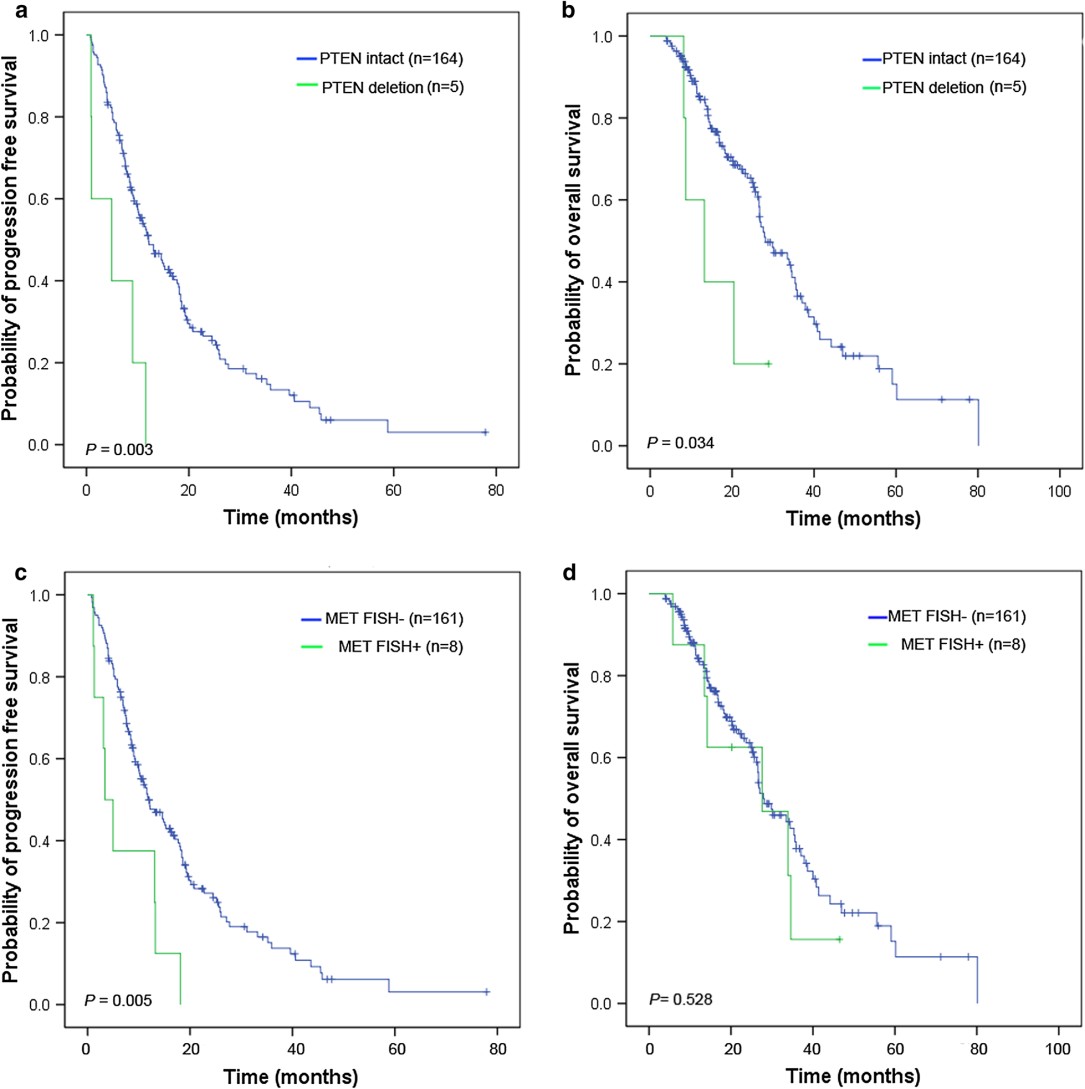
Kaplan-Meier curves of the progression-free survival (PFS) and overall survival (OS) for mutant-EGFR NSCLC patients with aberrations in PTEN and MET genes. PFS (**a**) and OS (**b**) were analyzed in the 169 EGFR-mutant NSCLC patients, according to PTEN copy number alterations. In the subgroups of EGFR-mutant NSCLC patients with MET FISH status, PFS (**c**) and OS (**d**) were analyzed. The survival rates were compared using the log-rank test

Among the 169 NSCLC patients with EGFR active mutations, 3 (1.8%) achieved CR, and 65 (38.5%) had PR, 87 (51.5%) had SD, and 14 (8.3%) had PD. EGFR-mutant patients having PTEN deletion had a shorter PFS and OS than those with intact PTEN (HR = 3.64, 95% CI = 1.47–9.00 for PFS, Fig. 3a, and HR = 2.86, 95% CI = 1.04–7.89 for OS, Fig. 3b). EGFR-mutant NSCLC patients with concurrent MET FISH(−) status had a superior PFS as compared to those with MET FISH(+) status (HR = 2.69, 95% CI = 1.30–5.54, Fig. 3c), whereas no significant difference was observed in the OS analysis for these subgroups. The aberrant alterations of BIM, PIK3CA, and IGF1R had no effect on PFS and OS in EGFR-mutant NSCLC patients (Additional file 5: Figure S4). No statistically significant differences in ORR and DCR among the groups were observed when stratified by the status of driver genes in the EGFR-mutant patients (Additional file 6: Table S2). A Cox proportional regression model revealed that PTEN deletion (HR = 4.29, 95% CI = 1.72–10.70), PTEN low expression (HR = 1.96, 95% CI = 1.22-3.13) and MET FISH(+) (HR = 2.83,95% CI = 1.37–5.86) remained strong and independent predictors for PFS in patients with EGFR-TKIs treatment after adjustment for multiple factors (Table 3). There were no patients harboring ALK apart and KRAS mutations in the mutant EGFR subgroup.

**Table 3:** Univariate and multivariate associations of EGFR mutation and FISH status, clinicopathological characteristics, and status of other seven genes with PFS and OS in the 169 NSCLC patients with EGFR mutation

Variable	PFS						OS					
	Univariate			Multivariate			Univariate			Multivariate		
	HR	95% CI	*P*	HR	95% CI	*P*	HR	95% CI	*P*	HR	95% CI	*P*
Age												
≤ 60	1						1					
> 60	1.07	0.74–1.54	0.721				0.77	0.49–1.22	0.266			
Gender												
Male	1						1					
Female	0.75	0.53–1.06	0.101				0.80	0.52–1.24	0.311			
Smoking status												
Smoking	1						1					
No smoking	0.69	0.47–1.03	0.066				0.71	0.44–1.14	0.157			
Histology												
Non-ADC	1						1					
ADC	0.84	0.47–1.49	0.539				0.75	0.37–1.52	0.424			
Differentiation												
High to medium	1						1					
Low	0.81	0.57–1.15	0.237				0.93	0.60–1.44	0.751			
Stage												
Recurrent	1						1					
IIIB and IV	0.91	0.63–1.32	0.628				0.92	0.59–1.45	0.730			
Line of TKI therapy												
2, 3	1						1					
1	0.94	0.66–1.35	0.734				1.01	0.65–1.58	0.954			
ECOG PS												
2	1						1			1		
0, 1	0.65	0.38–1.13	0.129				0.45	0.23-0.87	0.017	0.44	0.23-0.85	0.015
BIM												
Wt	1						1					
Deletion	1.37	0.79-2.35	0.261				1.69	0.91–3.13	0.099			
PIK3CA mutation												
Wt	1						1					
Mt	1.35	0.43–4.26	0.610				1.51	0.47–4.81	0.491			
PTEN copy number												
Intact	1			1.00			1					
Deletion	3.64	1.47–9.00	0.005	4.29	1.72–10.72	0.002	2.86	1.04–7.89	0.042	2.97	1.07-8.20	0.036
PTEN expression												
Normal	1			1			1					
Low expression	1.87	1.17-2.98	0.009	1.96	1.22–3.13	0.005	1.37	0.77–2.44	0.291			
MET copy number												
FISH−	1			1			1					
FISH+	2.69	1.30–5.54	0.008	2.83	1.37–5.86	0.005	1.31	0.57–3.01	0.529			
MET expression												
MAb−	1						1					
MAb+	1.02	0.68–1.55	0.913				0.67	0.36–1.25	0.213			
MET expression												
H-score−	1						1					
H-score+	1.36	0.95–1.95	0.692				1.24	0.79–1.96	0.348			
IGF1R copy number												
FISH−	1						1					
FISH+	0.35	0.05–2.48	0.291				1.14	0.16–8.32	0.893			
IGF1R expression												
IHC−	1						1					
IHC+	1.25	0.70–2.23	0.450				1.59	0.86–2.95	0.141			

*ADC* adenocarcinoma, *ECOG* Eastern Cooperative Oncology group, *TKI* tyrosine kinase inhibitors, *mt* mutation, *wt* wild-type, *FISH* fluorescent in situ hybridization, *HR* hazard ratio, *CI* confidence interval

## Discussion

To investigate the possible mechanisms underlying the primary resistance to EGFR-TKIs, we identified the aberrant alterations of 8 driver genes, and then analyzed the association between these genetic alterations and survival in a cohort of 416 consecutive NSCLC patients. We found that several genetic alterations, PTEN loss/deletion, and MET copy number gains had potentially underlying resistant genes in mutant-EGFR patients. A combined analysis of multiple genes showed that the greater the number of genetic alterations detected simultaneously, the poorer the PFS of the NSCLC patients were. The findings of this study could provide practical considerations for the EGFR-TKI treatment in the genotype-oriented treatment era.

Few large clinical trials have identified that NSCLC patients harboring sensitive mutations in EGFR were highly responsive to EGFR-TKIs [26, 27], but their findings were concordant with those of this study. However, clinical resistance inevitably exists in patients receiving EGFR-TKIs. There were few driver genes reported to be related to the primary resistance in lung cancer. A recent investigation showed that the nuclear accumulation of IGF1R in lung cancer contributed to EGFR-TKIs resistance observed in vivo and in vitro [28]. Choi et al. have reported that the combined inhibition of EGFR and IGF1R signaling pathways could induce the cell cycle arrest in NSCLC cell lines probably due to the EGFR pathway activated by IGF1R-mediated AKT bypass activation, which might lead to the primary resistance in NSCLC cell lines [29]. Several investigations also revealed that the interaction of EGFR and IGF1R may result in resistance to EGFR-TKIs in glioma [30] and breast and prostate cancers [31]. In a previous study on the efficacy of gefitinib, patients with IGF1R high expression had a favorable OS, whereas it had no predictable role in the aspect of the evaluation of gefitinib efficacy and PFS [32]. In our study, we found that IGF1R FISH+ was significantly associated with poorer PFS, but IGF1R high expression tended to be a risk factor for poorer PFS. This suggests that different antibodies and/or IHC scoring method that were applied in different populations could contribute to this observed inconsistency and that the prognostic role of IGFR-1 expression in patients not exposed to TKIs should be evaluated further. We also demonstrated the parameter for the determination of IGF1R FISH+ predicting poorer PFS, which could be implemented in the clinic to stratify NSCLC patients for specific treatments.

The BIM deletion polymorphism had been identified as a predictive marker for EGFR-TKIs treatment [24]. A previous study identified that an approximately 2900-bp deletion polymorphism of BIM could deregulate the proapoptotic function of the BIM protein, consequently inducing primary resistance in chronic myelogenous leukemia (CML) and NSCLC cell lines, and could predict a poor response to the EGFR-TKIs treatment [24]. However, a study by Lee et al. indicated that there was no role of BIM deletion observed in EGFR-TKIs treated patients [25]. In our cohort (n = 416), BIM deletion showed no effect on the PFS, ORR or DCR in patients receiving EGFR-TKIs. These apparently contradictory findings should be integrated into future studies. In one study by Sos et al. the authors demonstrated that PTEN loss could contribute to erlotinib resistance in EGFR-mutant NSCLC patients [33]. A mathematical modeling simulation denoted that PTEN loss resulted in an increased expression of AKT, which was related to EGFR-TKIs resistance [34]. The result of this present study was consistent with these studies, indicating that the loss/deletions of PTEN could cause primary resistance to TKIs in NSCLC patients.

In the combined analysis of gene expression, copy number, and mutations in all of the 416 patients and 169 mutant EGFR patients who exhibited possible primary resistance, we found that 336 NSCLC patients (80.0%) carried at least one RG alteration (ALK, KRAS, BIM, PIK3CA, MET, IGF1R, and PTEN), and 35 patients (20.7%) harbored coexisting genetic alterations in the EGFR signaling pathway (KRAS, PIK3CA, and PTEN). The survival analyses showed that EGFR-mutant patients with aberrant alterations in these genes had significantly poorer PFS than those without aberrant alterations, which could explain the primary resistance observed in our cohort. Several studies have reported that multiple genes concomitantly contribute to primary resistance. Two recent studies in East Asian populations reported that up to 10% of NSCLC patients with ALK fusion also had EGFR mutations [35, 36]. Previous studies have determined that EGFR T790M and MET amplification could coexist in a minor population of NSCLC cells before exposure to EGFR-TKIs [37, 38]. Lee et al. [25]. reported that 11 out of their investigated 197 mutant-EGFR NSCLC patients receiving TKIs exhibited primary resistance, and there were 3 patients who harbored either EGFR T790M, MET amplification, or ALK rearrangement. It was presumed that the coexistence of cancer-related genes might be an attribute to the mechanism of primary resistance. In an analysis of 11 patients with primary resistance, Kim et al. [12] demonstrated that the ORR in patients with EGFR signaling pathway mutations (PIK3CA, PTEN, AKT, STK11) was lower than in those without such alterations (14.5% vs. 63.6%). Studies on Spanish and Italian NSCLC patients identified that driver mutations, such as EGFR, KRAS, and PIK3CA and ALK rearrangement, could coexist in these patients, and that targeted treatment might not be as effective in patients with these coexisting mutations [39]. These studies suggested that the clonal selection of these cells during EGFR-TKI treatment resulted in TKI resistance.

Despite the interesting findings of this study, there are also some limitations that may be of concern. First, several genes, such as TP53 and RB1, which may be associated with the efficacy of EGFR-TKIs, were not be tested in this study. Second, the tested samples were all obtained at the time of primary diagnosis, and the second biopsies were not performed before EGFR-TKIs therapy, so the genetic status may have changed with the recurrence or metastasis of the tumors. Lastly, further prospective trials are warranted to confirm the effect of the genetic alterations in the driver genes on the efficacy of the EGFR-TKIs in NSCLC patients. Currently, comprehensive molecular detection in clinic would be the combination of high throughput sequencing and routine pathological techniques (such as FISH and IHC) based on the key genes (such as PTEN, MET, IGF1R, and et al.). On one hand, we can find the relevant factors to predict the efficacy of EGFR-TKIs; and on another hand, we can clearly identify the true status of genes to improve the selection of precise targeted agents.

## Conclusions

In this study, we identified that PTEN loss and increased MET copy number may predict an unfavorable survival in mutated-EGFR patients. The coexistence of genetic alterations in driver genes, indicating the cell subclone selection, may better explain the primary resistance to EGFR-TKIs.

## Additional files

**Additional file 1: Figure S1.** Representative images for fluorescence in situ hybridization (FISH) (×1000) analysis and staining of immunohistochemistry (IHC) (×200) for the different changes of seven driver genes in NSCLC patients. (a-b) FISH images of ALK wild and apart in patients. a, ALK break-apart signals by FISH with an isolated orange signal pattern was indicated as an ALK wild status; b, ALK break-apart signals by FISH with a split orange and green signal pattern was indicated as an ALK apart status. (c-f) FISH images of PTEN status in NSCLC patients. c, PTEN intact cases showed two CEP10 signals and two PTEN signal in tumor cells; d-e, The representative cases for PTEN homozygous deletion displayed PTEN/CEP10 ratio = 0.63 with two CEP10 signals and one PTEN signal in 80% of nuclei and PTEN/CEP10 ratio = 0.17 with two CEP10 signals and no PTEN signal in 70% of nuclei in NSCLC patients, respectively; f, One CEP10 and one PTEN signal in 70% of nuclei is considered as whole chromosome 10 deletion. (g-i) The representative images of FISH for MET status in NSCLC patients. g, MET FISH- was identified as disomy; h, MET CNV = 5.8 and high polysomy ≥ 4 copies in 67% of tumor cells were considered as MET FISH+; i, MET CNV = 12/chr7 CNV = 5.4 with ratio = 2.22 is determined as MET amplification; (j-k) The detection of IGF1R status using FISH in NSCLC patients. j, IGF1R FISH- was identified as disomy; k, Polysomy ≥ 4 copies in 70% of tumor cells and IGF1R CNV = 7.5/chr5 CNV = 4.2 were considered as IGF1R FISH + . (l-m) The detection of PTEN expression using IHC in patients. l, PTEN IHC staining shows cytoplasma of NSCLC tumor cell; m, PTEN low expression or loss were considered as negative staining. (n-p) The representative IHC images for MET expression in patients. n, No expression of MET was indicated as MET IHC−; o, H-score = 170 was considered as MET Mab− and MET IHC−; p, H-score = 310 was identified as MET Mab+ and MET IHC+. (q-s) The detection of IGF1R expression using IHC. q, no expression was considered as IGF1R IHC−; IGF1R IHC+ includes H-score = 135 (r) and H-score = 330 (s).

**Additional file 2: Table S1.** Clinical characteristcs of 416 NSCLC patients harboring alterations of seven driver genes.

**Additional file 3: Figure S2.** Kaplan–Meier curves of progression-free survival (PFS) for NSCLC patients with aberrant alterations of each gene. In the total of 416 NSCLC patients, PFS (a) was analyzed according to the EGFR mutation status; (b) was analyzed according to the EGFR FISH±; (c) was analyzed according to the BIM mutation status; (d) was analyzed according to the ALK wild/apart status; (e) was analyzed according to the KRAS mutation status; (f) was analyzed according to the PIK3CA mutation status; (g) was analyzed according to the PTEN intact/deletion status; (h) was analyzed according to the PTEN expression status; (i) was analyzed according to the MET FISH± status; (j) was analyzed according to the MET Mab± status; (k) was analyzed according to the MET H-score± status; (l) was analyzed according to the IGF1R FISH± status; (m) was analyzed according to the IGF1R IHC± status. The survival rates were compared using the log-rank test.

**Additional file 4: Figure S3.** Kaplan–Meier curves of overall survival (OS) for NSCLC patients with aberrant alterations of each gene. In the total of 416 NSCLC patients, OS (a) was analyzed according to the EGFR mutation status; (b) was analyzed according to the EGFR FISH±; (c) was analyzed according to the BIM mutation status; (d) was analyzed according to the ALK wild/apart status; (e) was analyzed according to the KRAS mutation status; (f) was analyzed according to the PIK3CA mutation status; (g) was analyzed according to the PTEN intact/deletion status; (h) was analyzed according to the PTEN expression status; (i) was analyzed according to the MET FISH± status; (j) was analyzed according to the MET Mab± status; (k) was analyzed according to the MET H-score± status; (l) was analyzed according to the IGF1R FISH± status; (m) was analyzed according to the IGF1R IHC± status. The survival rates were compared using the log-rank test.

**Additional file 5: Figure S4.** Kaplan–Meier curves of progression-free survival (PFS) and overall survival (OS) for 169 mutant-EGFR NSCLC patients with aberrant alterations of each gene. PFS (a) and OS (b) were analyzed according to the BIM mutation status; PFS (c) and OS (d) were analyzed according to the PIK3CA mutation status; PFS (e) and OS (f) were analyzed according to the PTEN expression status; PFS (g) and OS (h) were analyzed according to the MET Mab± status; PFS (i) and OS (j) were analyzed according to the MET H-score± status; PFS (k) and OS (l) were analyzed according to the IGF1R FISH± status; PFS (m) and OS (n) were analyzed according to the IGF1R IHC± status. The survival rates were compared using the log-rank test.

**Additional file 6: Table S2.** The association of ORR and non-ORR, DCR and non-DCR with the status of five cocurrence driver gens in the patients with EGFR-mutation, respectively.

## Abbreviations

NSCLC: non-small-cell lung cancer
EGFR: epidermal growth factor receptor
TKI: tyrosine kinase inhibitor
PFS: progression-free survival
OS: overall survival
ADC: adenocarcinoma
PD: progressive disease
SD: stable disease
PR: partial response
CR: complete response
ORR: objective response rate
DCR: disease control rate
CI: confidence interval
KRAS: Kirsten rate sarcoma viral oncogene homolog
PIK3CA: phosphatidylinositol-4,5-bisphosphate 3-kinase catalytic subunit alpha
PTEN: phosphatase and tensin homologue
MET: MET proto-oncogene, receptor tyrosine kinase
IGF1R: insulin-like growth factor 1 receptor
ALK: anaplastic lymphoma kinase
BIM: BCL2 like 11
ROS1: ROS proto-oncogene 1
IHC: immunohistochemistry
FISH: fluorescence in situ hybridization
HR: hazard ratio
