# Supplementary figures and images for: Identification of genetic alterations associated with primary resistance to EGFR-TKIs in advanced non-small-cell lung cancer patients with EGFR sensitive mutations
Source: Cancer Commun (Lond). 2019 Mar 2;39:7. doi: 10.1186/s40880-019-0354-z (PMC6397445; doi:10.1186/s40880-019-0354-z)

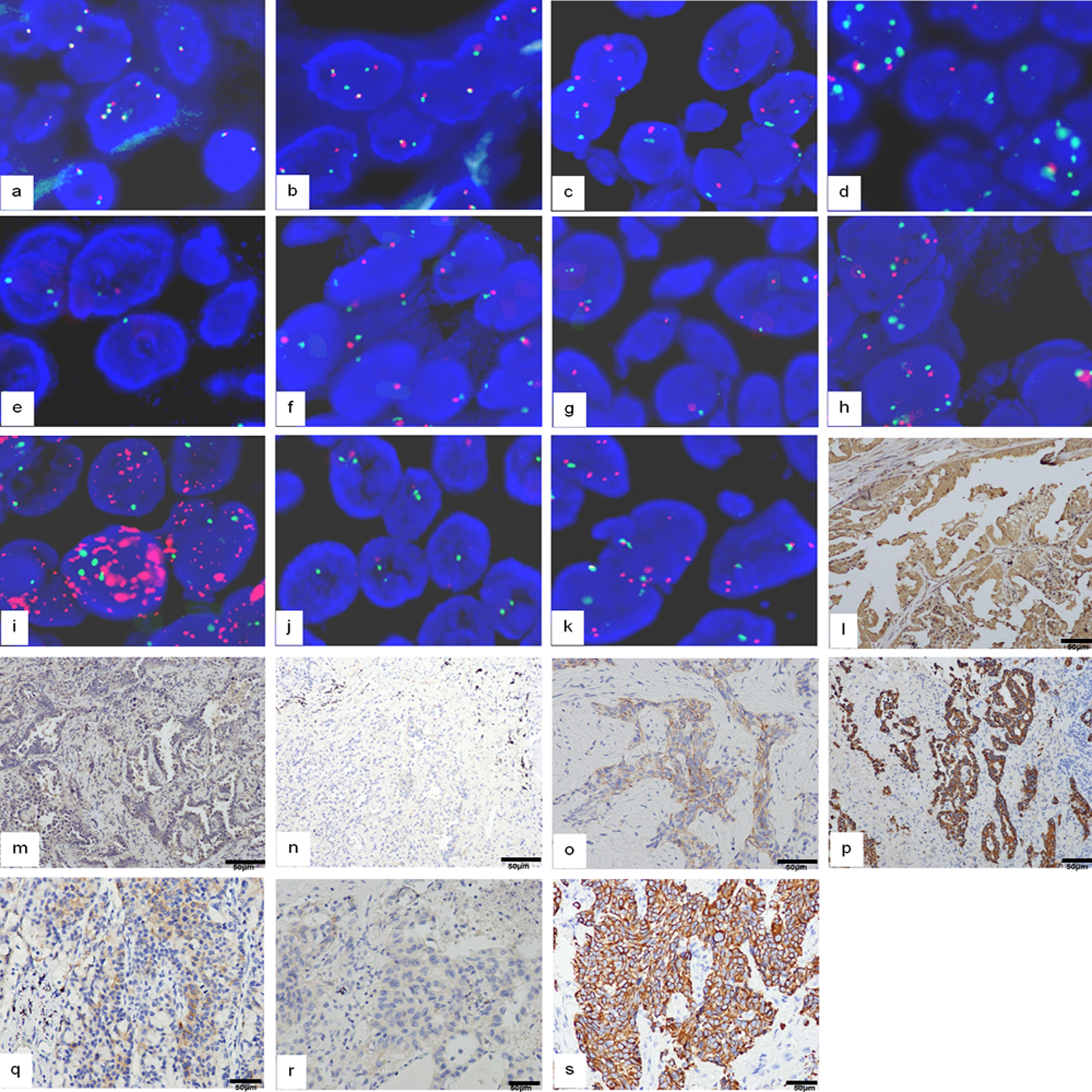

Supplement: Supplementary file 1 — Additional file 1: Figure S1. Representative images for fluorescence in situ hybridization (FISH) (×1000) analysis and staining of immunohistochemistry (IHC) (×200) for the different changes of seven driver genes in NSCLC patients. (a-b) FISH images of ALK wild and apart in patients. a, ALK break-apart signals by FISH with an isolated orange signal pattern was indicated as an ALK wild status; b, ALK break-apart signals by FISH with a split orange and green signal pattern was indicated as an ALK apart status. (c-f) FISH images of PTEN status in NSCLC patients. c, PTEN intact cases showed two CEP10 signals and two PTEN signal in tumor cells; d-e, The representative cases for PTEN homozygous deletion displayed PTEN/CEP10 ratio = 0.63 with two CEP10 signals and one PTEN signal in 80% of nuclei and PTEN/CEP10 ratio = 0.17 with two CEP10 signals and no PTEN signal in 70% of nuclei in NSCLC patients, respectively; f, One CEP10 and one PTEN signal in 70% of nuclei is considered as whole chromosome 10 deletion. (g-i) The representative images of FISH for MET status in NSCLC patients. g, MET FISH- was identified as disomy; h, MET CNV = 5.8 and high polysomy ≥ 4 copies in 67% of tumor cells were considered as MET FISH+; i, MET CNV = 12/chr7 CNV = 5.4 with ratio = 2.22 is determined as MET amplification; (j-k) The detection of IGF1R status using FISH in NSCLC patients. j, IGF1R FISH- was identified as disomy; k, Polysomy ≥ 4 copies in 70% of tumor cells and IGF1R CNV = 7.5/chr5 CNV = 4.2 were considered as IGF1R FISH + . (l-m) The detection of PTEN expression using IHC in patients. l, PTEN IHC staining shows cytoplasma of NSCLC tumor cell; m, PTEN low expression or loss were considered as negative staining. (n-p) The representative IHC images for MET expression in patients. n, No expression of MET was indicated as MET IHC−; o, H-score = 170 was considered as MET Mab− and MET IHC−; p, H-score = 310 was identified as MET Mab+ and MET IHC+. (q-s) The detection of IGF1R expression [file 40880_2019_354_MOESM1_ESM.tif]

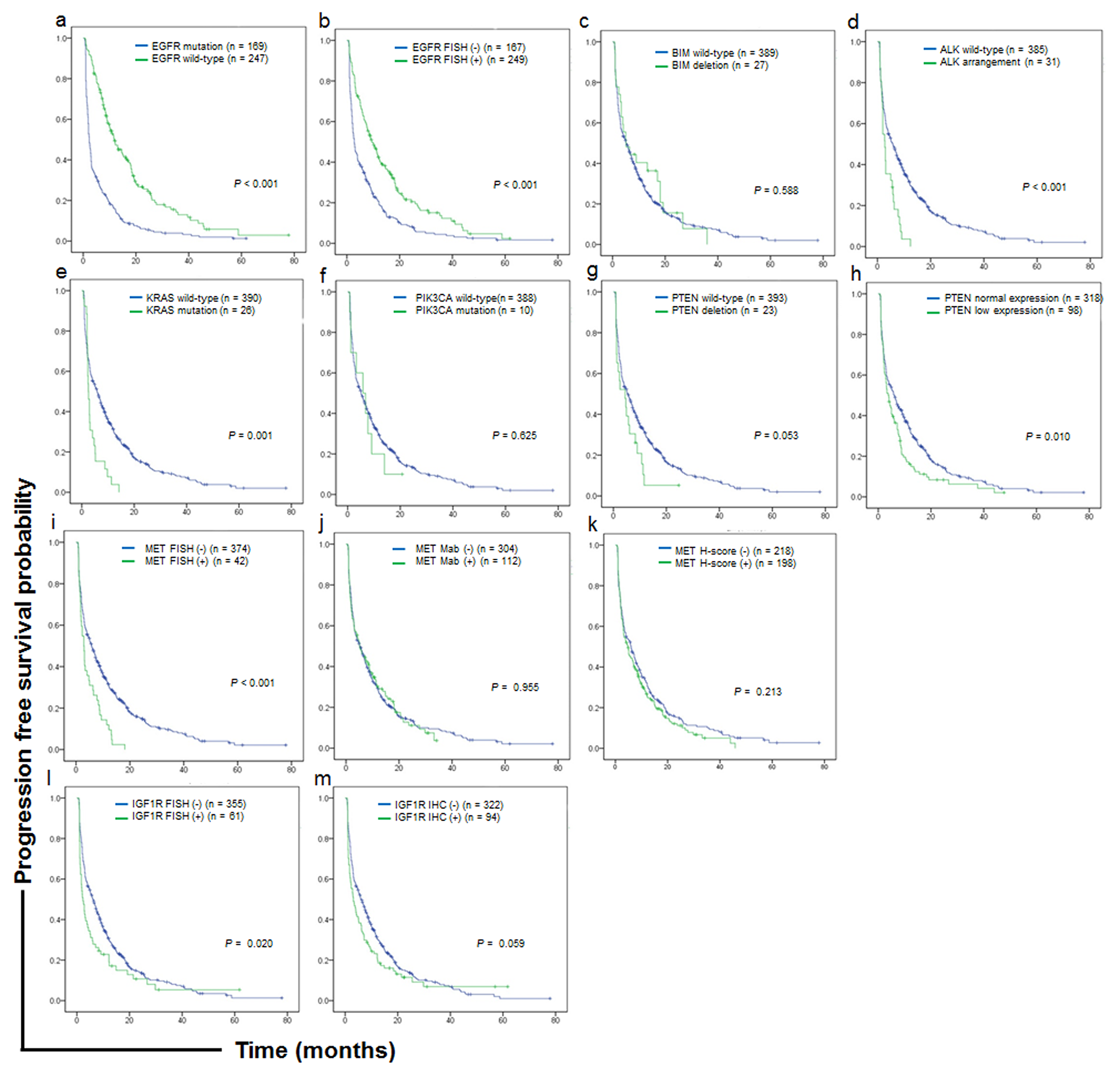

Supplement: Supplementary file 3 — Additional file 3: Figure S2. Kaplan–Meier curves of progression-free survival (PFS) for NSCLC patients with aberrant alterations of each gene. In the total of 416 NSCLC patients, PFS (a) was analyzed according to the EGFR mutation status; (b) was analyzed according to the EGFR FISH±; (c) was analyzed according to the BIM mutation status; (d) was analyzed according to the ALK wild/apart status; (e) was analyzed according to the KRAS mutation status; (f) was analyzed according to the PIK3CA mutation status; (g) was analyzed according to the PTEN intact/deletion status; (h) was analyzed according to the PTEN expression status; (i) was analyzed according to the MET FISH± status; (j) was analyzed according to the MET Mab± status; (k) was analyzed according to the MET H-score± status; (l) was analyzed according to the IGF1R FISH± status; (m) was analyzed according to the IGF1R IHC± status. The survival rates were compared using the log-rank test. [file 40880_2019_354_MOESM3_ESM.tif]

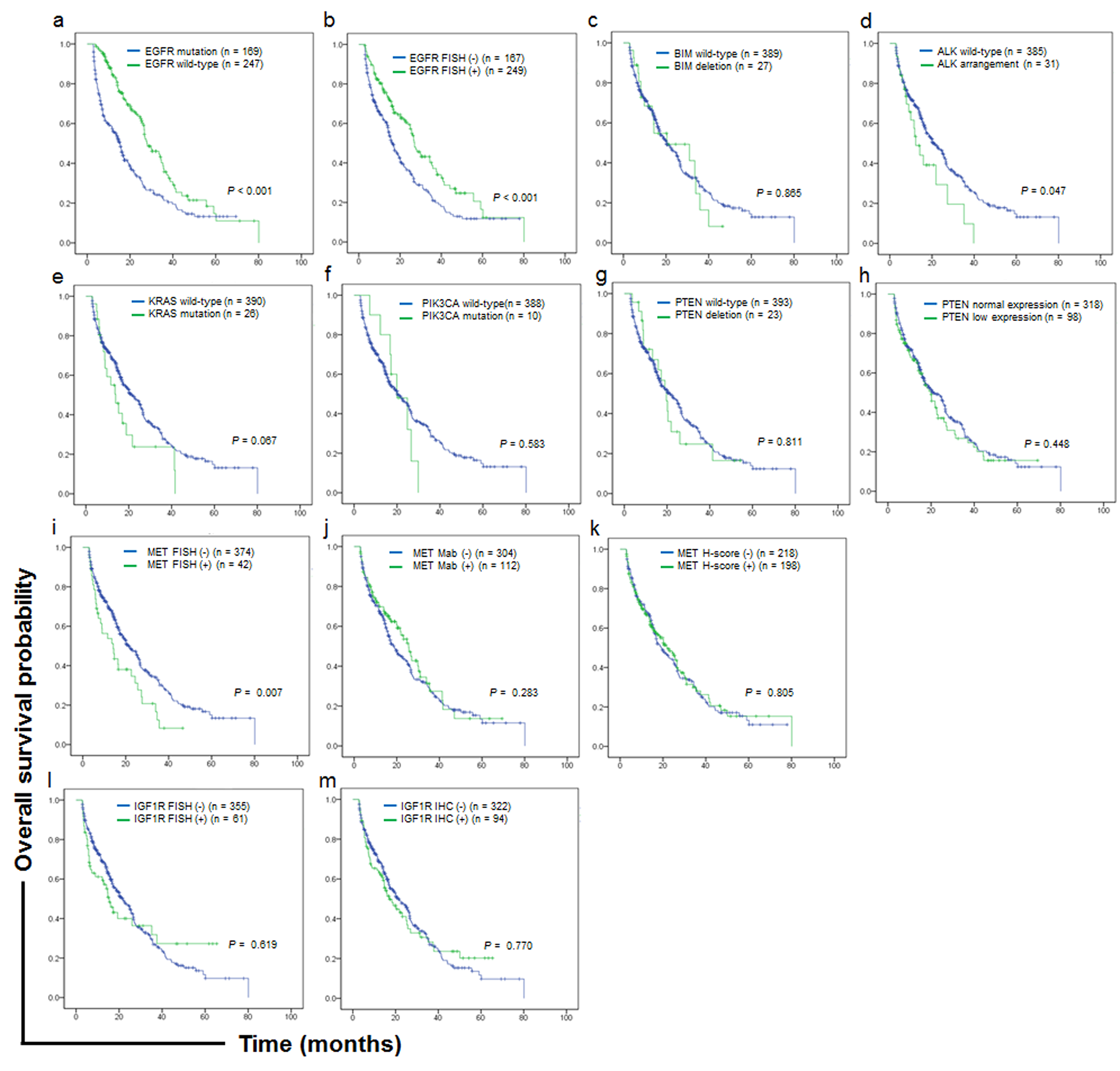

Supplement: Supplementary file 4 — Additional file 4: Figure S3. Kaplan–Meier curves of overall survival (OS) for NSCLC patients with aberrant alterations of each gene. In the total of 416 NSCLC patients, OS (a) was analyzed according to the EGFR mutation status; (b) was analyzed according to the EGFR FISH±; (c) was analyzed according to the BIM mutation status; (d) was analyzed according to the ALK wild/apart status; (e) was analyzed according to the KRAS mutation status; (f) was analyzed according to the PIK3CA mutation status; (g) was analyzed according to the PTEN intact/deletion status; (h) was analyzed according to the PTEN expression status; (i) was analyzed according to the MET FISH± status; (j) was analyzed according to the MET Mab± status; (k) was analyzed according to the MET H-score± status; (l) was analyzed according to the IGF1R FISH± status; (m) was analyzed according to the IGF1R IHC± status. The survival rates were compared using the log-rank test. [file 40880_2019_354_MOESM4_ESM.tif]

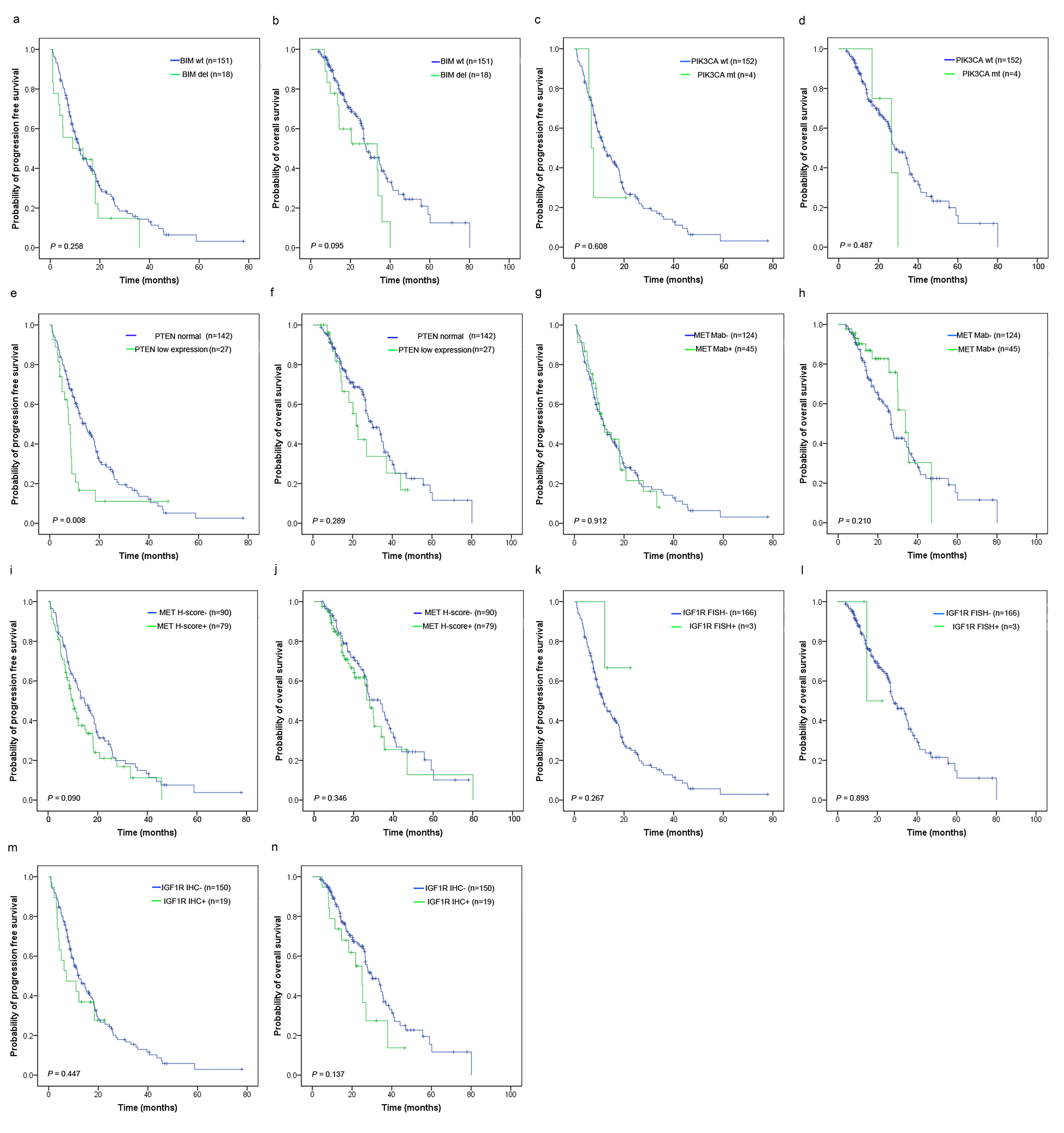

Supplement: Supplementary file 5 — Additional file 5: Figure S4. Kaplan–Meier curves of progression-free survival (PFS) and overall survival (OS) for 169 mutant-EGFR NSCLC patients with aberrant alterations of each gene. PFS (a) and OS (b) were analyzed according to the BIM mutation status; PFS (c) and OS (d) were analyzed according to the PIK3CA mutation status; PFS (e) and OS (f) were analyzed according to the PTEN expression status; PFS (g) and OS (h) were analyzed according to the MET Mab± status; PFS (i) and OS (j) were analyzed according to the MET H-score± status; PFS (k) and OS (l) were analyzed according to the IGF1R FISH± status; PFS (m) and OS (n) were analyzed according to the IGF1R IHC± status. The survival rates were compared using the log-rank test. [file 40880_2019_354_MOESM5_ESM.tif]
